# Barriers, Attitudes, and Influences Towards Dietary Intake Amongst Elite Rugby Union Players

**DOI:** 10.3389/fspor.2021.789452

**Published:** 2021-12-20

**Authors:** A. M. Sharples, Stuart D. Galloway, D. Baker, Brett Smith, Katherine Black

**Affiliations:** ^1^Faculty of Health Sciences and Sport, University of Stirling, Stirling, United Kingdom; ^2^Department of Human Nutrition, University of Otago, Dunedin, New Zealand; ^3^Department of Sports and Medicine, Axis Sports Medicine, Auckland, New Zealand; ^4^Te Huataki Waiora School of Health, University of Waikato, Hamilton, New Zealand

**Keywords:** athletes, food insecurity, diet, body composition, development

## Abstract

**Background:** Dietary intakes can impact an athletes health and performance. Although evidence exists about what an athlete should eat, an athlete's nutritional intake is influenced by many factors. The limited research available suggests the main barriers preventing optimal nutritional intakes reported by athletes are lack of time, food accessibility, poor cooking skills, costs, taste, and time spent in “off-season.” Although these factors have been shown to influence dietary intake they remain relatively unexplored in Rugby Union. This study aimed to describe the nutritional influences on dietary intake amongst Rugby Union player's.

**Methods:** This was a qualitative study utilising in person individual interviews with all participants. Participants were Rugby Union players (*n* = 30) for either a Super Rugby franchise or one of their development squads in New Zealand. Participant's undertook recorded face to face interviews, which were later transcribed. A thematic approach was used to code the transcripts by the primary coder and the themes were subsequently evaluated by the research team.

**Results:** Childhood upbringing, organisational skills, time and food security also emerged as barriers. Body composition and sport nutrition knowledge emerged as both barriers and enablers to nutritional intake. Influence on performance was an enabler to optimal dietary intake. Fully professional rugby players have access to dietitians, whereas development and semi-professional rugby players only have limited if any access, and they were more likely to seek nutritional information via social media.

**Conclusion:** This study suggests a need for greater nutrition education at developmental levels with an emphasis on affordable food choices, meal planning and skills for interpreting online nutrition information.

## Introduction

Optimising nutritional intakes is an important component to maximise performance, promote physiological adaptations to training, assist in recovery, alter body composition and protect overall health of athletes (Beck et al., 2015; Devlin and Belski, 2015; Black et al., 2018). Studies have investigated nutritional requirements, intakes and knowledge of Rugby Union players (Holway and Spriet, 2011; Bradley et al., 2015a,b; Black et al., 2018). This research has shown that players follow a low carbohydrate high protein diet, which may not be optimal (Holway and Spriet, 2011; Bradley et al., 2015a,b; Black et al., 2018).

An athlete's nutritional intake can also be influenced by many sociological, cultural and physiological factors (Sobal and Bisogni, 2009). Given the importance of nutrition to an athlete's ability to perform, train, recover and optimise body composition, an understanding of these influences on intake is required when providing nutritional advice (Sobal and Bisogni, 2009). However, there has been very limited research surrounding influences on dietary intakes. Heaney (2011) explored influences on dietary practises of elite-level athletes, coaches and sports dietitians from the Australian Institute of Sport (AIS). They identified that the barriers to healthy eating included lack of time for food preparation, financial limitations, inadequate cooking skills and difficulty with living arrangements. They also reported that concerns around body shape due to societal pressures influenced dietary habits. They concluded that these themes could be implemented in educational programs surrounding food choices. Similar barriers, i.e., lack of time, poor cooking skills, and food costs, were also reported in a review of influences on dietary intakes by Birkenhead and Slater (2015). They also noted other barriers such as access to appropriate food for sport, taste, hedonic hunger, “off-season,” and compensating for increased energy expenditure with greater food intake.

In contrast, enablers to appropriate food choices have been reported to include higher income, nutrition knowledge, belief that nutrition is important for performance, body composition goals, and family support (Worsley, 2002; Wardle et al., 2004; Heaney, 2011; Birkenhead and Slater, 2015).

There is currently only one study which has explored the perceptions and determinants of eating for health and performance in Rugby Union players, however it was amongst adolescent players (Stokes et al., 2018). They showed that both adolescent and sport-specific determinants influenced the food choices of participants. Those relevant to adolescence included, the influence of significant others such as peers and family, taste, cost, convenience and food availability. Sports-specific determinants revolved around the desire to enhance performance, motivation, and team culture. Both adolescent and sport-specific interrelated factors were identified as media (mainstream and social media), physical appearance, and feeling good. Whether the same influences are seen amongst elite rugby union players is unclear. Knowledge of the influences on dietary intakes could help inform dietary interventions in elite rugby union environments.

Therefore, with no documented studies exploring the barriers, influences and attitudes affecting the food choices and intake of elite Rugby Union players, this study aimed to describe perceived influences on dietary intakes amongst New Zealand Rugby Union players.

## Materials and Methods

Thirty male Rugby Union players, aged 17–28 years were recruited. To participate they had to be currently playing with a Super Rugby Franchise (professional), Provincial Rugby squad (semi-professional), or playing for developmental rugby for one of these teams, 30 players volunteered and completed all aspects of the study (see Table 1) for demographics. All players provided informed written consent prior to participating and the research was conducted in accordance with the Declaration of Helsinki. Ethical approval was obtained through by both The University of Otago (reference: 17/168) and The University of Stirling Ethics Committees (reference: NICR 18/19).

**Table 1:** Demographic and body composition of participants.

	***n***	**%**	
**Level of play**			
Super rugby	19	63	
Provincial rugby	8	27	
Super rugby development	3	10	
**Ethnicity**			
New Zealand Māori	8	27	
New Zealand European	8	27	
Samoan	8	27	
Tongan	5	17	
Other	1	2	
	**Mean**	**SD**	**Range**
**Age (years)**	22.5	2.6	17.0–28.3
**Body composition**			
Weight (kg)	103.4	17.1	79.0–140.0
Height (cm)	186.8	0.1	171.0–200.0
Sum 8 skinfold (mm)	86.0	25.4	47.8–143.9

The study used a cross-sectional observational design and participant's undertook a face to face interview about nutrition, upbringing and body composition. Whereby the general inductive approach was chosen as a well-accepted qualitative method that enabled the researchers to identify themes in relation to the research objectives (Thomas, 2006). Body mass (kg) and sum of eight skinfolds (mm) (sites: Biceps, Triceps, Subscapular, Iliac Crest, Supraspinale, Abdominal, Front Thigh, Mid Calf) were obtained from the team's dietitian, a level 1 anthropometrist, using standard International Association of Kinanthropometry (ISAK) protocols (Norton and Olds, 1996). All measures were taken in duplicate and the mean value used unless the difference in the two measures was >5% then a third measure was obtained and the median value used. In Rugby Union it is common to use weight (kg) and sum of eight skinfolds (mm) as indicators of body composition. Unfavourable body composition was determined by the performance team and was defined as those players ±2 kg away from their individualised weight goal and/or ±20 mm away from their individualised skinfold goal.

Following a search of the previous literature regarding influences on dietary intakes of athletes a list of interview questions were developed (Worsley, 2002; Wardle et al., 2004; Heaney, 2011; Birkenhead and Slater, 2015). These were reviewed by nutritionists working with elite athletes and rugby players and any recommendations were implemented. The interview questions were then piloted with a group of athletes to determine their interpretation and understanding of the questions. Following this modifications were made as needed.

Trained researchers (AS, KB, SC, JS) undertook individual face-to-face semi-structured interviews with the participants at the start of pre-season. These interviews started by the researches explaining the research and then asking the players about their rugby career to date, including clubs played for and playing position. Players were then asked about their nutrition knowledge including if they had ever studied a nutrition course, where they get their information about nutrition from, if they ever use social media to obtain nutrition or diet information and if so what information do they read and how do they use it. Information on their childhood began by asking them about their living situation when they were young including the number of people living in the house, asking them to describe their diet growing up including some typical meals, this was followed up with questions about how these dietary patterns tracked into adulthood and if there were any reasons or influences on their childhood diet. Players were asked to describe how they planned and shopped for food during a typical week, what influenced their decision to eat the foods they did, if they skipped any meals and if so why. Players were also asked about their beliefs in regard to importance of nutrition for their performance and the way they look. Finally players were asked about their current living situation and who does the majority of cooking in the household and if they are able to cook and if so what meals they are able to make. Interviews were audio recorded and observations taken during the interviews were taken to enrich the data. The interview themes (Table 2) developed by the research team were based on current research and clinical experience from sports dietitians/researchers of elite athletes (Heaney, 2011; Birkenhead and Slater, 2015). Participants were prompted to expand and explain responses and indicate how this impacted on their dietary intake, however care was taken not to lead participant responses. All interviews were audio recorded and lasted 20–50 min.

**Table 2:** Interview themes used to promote discussions about nutritional influences in elite rugby union players.

**Theme**	**Example question**
Playing career	Can you tell me a bit about your playing career so far i.e., club names, seasons with each club, playing positions and nutritional support available for example did they have a dietitian/nutritionist, nutrition talks if so who from?
Dietary intakes	Do you find yourself eating a lot late at night?
Nutrition knowledge	Do you ever look on the internet or social media for nutrition information? If so, where and for what type of information?
Nutrition beliefs	To you is your body composition important and why? Appearance, performance, confidence?
Childhood nutrition (Up to 16 years old)	What foods would you have as treats as a child? How frequently did you have them?
Cooking ability	What can you cook? Ask if from basic ingredients or ready-made meals/sauces.
Current living situation	Who do you live with at the moment? Who does the cooking?

Interviews were verbatim transcribed into word files by one researcher (AS). Transcripts underwent thematic analysis used to establish key themes within the data and develop a structural framework around the ideas. Initial coding and theme development was undertaken manually by one researcher (AS). Confirmation and consensus was obtained through discussion between the research team (AS, KB). Barriers were classified as factors that negatively influenced optimal dietary intake and attitudes as the participant's views towards optimal nutrition strategies. Whereas, enablers were classified as factors which positively affected dietary behaviours related to nutritional guidelines. Quotes were extracted to represent the themes identified, with selected word for word quotes presented in the results.

Participant characteristics were described using mean ± standard deviation (SD). Questionnaire data was reported as categorical data n (%). Thirty interviews was sufficient to provide thematic saturation (i.e., further interviews were unlikely to elicit new information).

## Results

**Figure 1:**
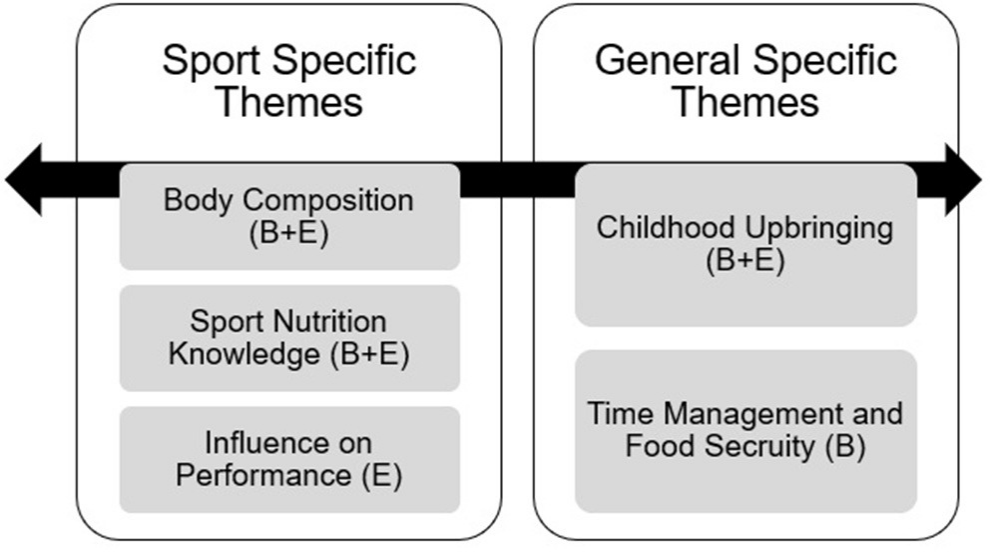
Sport and general specific themes from thematic analysis of elite RU player's nutrition interviews. E, Enabler; B, Barrier.

Each participant identified a number of barriers and perceived attitudes that they believed to influence their capability to eat an appropriate diet or maintain an optimal body composition. These main barriers and attitudes emerging from the interviews can be classified into sport specific and general specific themes (Figure 1). These themes are detailed in the following paragraphs.

Sport specific knowledge allows for participants to optimise nutrition strategies within and outside the training environment. Participants at provincial and development level lacked knowledge compared to those at Super Rugby Level, although academy squads did provide nutritional support.

“*They do give you a good idea about it, but probably at that age you're not really taking as much of an interest in that because that kind of isn't important” (Super Rugby player #2, discussing nutrition support at the academy level)*.

“*Yeah. I feel like I've got quite a bit of knowledge on eating healthy. Just because I'm obviously trying to gain weight all the time. So I've got to be, um have those meals prepared and whatnot.”(Super Rugby player #18)*

Of those (*n* = 8) who sought outside sources to get nutrition information none were contracted to a Super Rugby Franchise.

“*Yeah 100%” (Academy player #1)* in response to do you get your nutrition information from social media or the internet.

“*Usually just word of mouth, like players talking like I was on a different diet in France. I was eating whatever I want but all carbs and all sugar. I was eating before 12:00. And then from 12:00 onwards was either salads or straight proteins.” (Super Rugby player #18 talking about his time before his Super contract)*

Those at Super Rugby level had greater contact with Sports Dietitians, more nutrition information sessions and resources. In response to the question about seeking advice online or social media Super rugby players stated

“*I used to (Ed: follow advice on the internet), before I ever went through all that nutritional stuff. But not really, to be honest with you. Just, no, I'd rather go through the nutritionist that I'm with at the time.” (Super rugby player #5). “I'd just ask XXXX (ed: team nutritionist) for help” (Super rugby player #19)*.

Body composition was identified as an important factor to the dietary intake of participants.

“*I try to eat healthy just in general because I've always had issues with my skinfolds being a little bit too high” (Super Rugby player #13)*.

Of those interviewed 53% (*n* = 16) participants worried about their skinfolds. “*Uh, yeah. A lot I do worry about it. But sometimes I just when it comes to food I don't think of what it can do to me all the bad junk stuff.”* (Super rugby player #29 who worries about skinfolds a lot as they are always too high).

Consistency and planning was a theme from the interviews with the theme suggesting players who suggested that they struggled with body composition, “*Probably need to be more consistent with planning my meals on a Sunday or something for the week and just stuff like that”* (New signing at Super Rugby #27) and that they often “shop daily for food rather than plan,” or that if they try to plan they do not stick to their plans “*Well I've been told to do that (ed: stick to a weekly meal plan) but it's hard. It's not. I'm not the sort of person to do that because I go by everyday. I'll get up and I'll feel like this and that. Then the weekend I'll plan for something but then end up eating something else” (Player #16*). Whereas, players who were less concerned about their skinfolds as they usually had skinfold measures within the desired range stated the theme was more along the lines of planning and consistency “*I sort of try to plan, but I'm sort of more planned around my snacks because I feel like that's sort of where my main sort of gains will come from.”* (Super rugby player #28). “* Yeah usually at the start of the week, we'll do our food shop and then we'll plan sort of a few meals, and we might see what meat's on special or something like that. And then we'll have sort of regular breakfast stuff like porridge and basics. Lunch is probably a bit flexible… But yeah dinners are usually planned out for the week” (Player #15) “I sort of eat things like training and load. But I just eat around training sort of thing. So whatever I have on for that day. So if I've got a heavy day of training and work, then I'll probably eat more than what I would on a day that I wouldn't” (Academy player #4 who shopped weekly and usually had skinfolds close to their goal)*.

There was also an emerging relationship between body composition and appearance, with players responding to why they eat the way they do with “*Um yes I don't know. I think it's just so I can fit into the clothes I wanna wear and stuff like that.” (Player #19) “I think appearance is always, for boys, is always kind of a thing. For that muscle definition and things” (Player #13)*.

Body composition was also identified as being an enabler to healthy eating and optimising sport nutrition strategies. Participants identified that body composition was important to their performance and influenced their dietary intake. The relationship between body composition and performance in rugby is important for performance.

“*Oh, being the outside back, obviously I need to be fast, so I guess the more skinfolds, the slower I'm going to be. So probably need to get onto it.” (Player #17)*

“*Performance, yes because you don't want to be carrying around too much crap weight.” (Player #32)*

“*I'd say performance. If it was clear-cut that if I, I don't know, lost X amount of skinfolds or gained X amount of muscles, increase my performance, that would definitely be the huge motivating factor.” (Player#14)*.

“*Yeah. I think its pretty important (ed: body composition) for me because I've seen how easy it is to move and to when you're in shape. And I get told that a lot.” (Super rugby player #21)*

“*Yeah. Yeah. I would think so especially in my position because if I'm like fat, then I won't be fast So I think or playing in the backs you need to be relatively lean” (Player #30)*.

Childhood upbringing was a major theme raised by the participants as a barrier to both body composition and healthy eating habits. Childhood barriers involved family size (determined by the number of family members living in their house growing up), nutrition routine e.g., eating at the table, food costs and food availability. From the participant group, 53% (*n* = 16) grew up in families greater than five. Of this group 70% (*n* = 9) were of Pacific Island descent, 15% Maori (*n* = 2) and 15% (*n* = 2) NZ European. Family members included parents, siblings, grandparents, aunts & uncles, cousins and extended family. Of those participants who grew up in families greater than five, 81% (*n* = 13) thought they didn't eat well growing up.

In response to “*do you think you ate well growing up?” “No Uh, just whatever my parents could, um, afford. Yeah. Food that could feed our family” (Player #19)*.

Of those who grew up in families greater than five, 81% (*n* = 13) diets were restricted by budget while they were growing up. Players from large families stated “*Yeah. I think we ate minimal. We weren't that well off, so we. So it was definitely—your food was definitely restricted.” (Player #6)* And “*We just ate whatever was cheapest, like a lot of bread for sure, noodles, probably not the best stuff. Oh, eggs, yeah, a lot of eggs, and cereal, yeah, one-off big barbecues, but yeah. Mostly noodles was pretty good. Happy with that.” (Player #7) “We mainly just lived off bread and stuff. So just sort of ate quite a bit of bread actually” (Player #26 lived with eight and reported money was tight). “Um Yeah. Maybe every week (ed: rely on others for food). Sometimes, I just have to have toast or something for dinner, so. That was just like maybe every couple of weeks, or every fortnight I guess” (Player #17 family of six and struggled to gain weight suggested by academy teams)*.

Childhood food routine was considered a determining factor in dietary habits that were carried into adulthood. “*I think so. I think, because I didn't really have much, having treats was like a I don't know–we didn't have it much, so when we, it would be hidden, and then when you'd get it, you'd be like, oh, happy as. And so I think that's why now I eat so much because I can get it whenever I want. When I was younger, it was just maybe once a week or something” (Player #17). “I think they have with the junk food side. And like sometimes reaching for the wrong food. But I suppose I've sort of broken the habit of continuously eating bad food. I don't reach for it as much. Um like But the tendency's still there, I suppose (Player #14)*.

Sayings around dinner table during childhood that were carried onto adulthood.

“*That's one I grew up with mum would always expect, Finish everything on the plate.” … “I would say so. I always finish my plate.” (Player #16)*

“*Like I'm pretty sure you had to eat, like, your vegetables and before you could have pudding… Yeah. Always, yeah Always eat my vegetables.” (Player #25)*

Those who skipped breakfast and lunches during childhood were more likely to skip meals during adulthood. For example, one player who reported that they did not consume breakfast everyday in response to questioning about habits from childhood which still exist…

“*Yes. Not really eating breakfast. Not wasting food. Habits that've been built since I was little” (Player #10)*.

The skipping of meals can lead to missed opportunities for nutrition intake as well as increased risk of bingeing at later meals.

Time was an emerging theme as a barrier for athletes in eating an appropriate diet and is strongly linked to other barriers such as food security and cost. The participant group of thirty Rugby Union players competing at an elite level, typically trained up to 30 h plus per week in addition to balancing study, work and family commitments. Time management including consistency, preparation time and discipline were identified by 57% (*n* = 17) of participant's as the biggest challenge to eating a healthy diet. “*Um, what prevents me from changing the way that I, I eat? I think just probably just not being actually I don't know. Yeah. Yeah, I think unmotivation or just time management. Yeah” (Super Rugby player #22)*. Food security and food cost also emerged as themes that inhibited healthy food intakes and/or habits.

“*Yeah, try to eat healthy. Just sort of, when I can eat really, and because of work and stuff at the moment and then sort of money wise.”* (academy player #26 working night shifts)

“*Not in the past, but probably where I am now, I'm a replacement player here, so I don't have anything long-term.”* (Super rugby #15 replacement player in response to being questioned about money influences on diet).

## Discussion

Participant's in the current study report barriers to optimal nutritional intake which could be classed as either sport specific or general themes; childhood, body composition, nutrition knowledge, time management and food security, which is in line with previous research studies in athletes (Heaney, 2011; Birkenhead and Slater, 2015; Stokes et al., 2018; Bentley et al., 2021). Highlighting that factors aside from nutritional needs should be considered when providing information to athletes.

The influence of childhood upbringing as both a barrier and enhancer to dietary habits is interesting and shows the need for greater nutritional support at the developmental level. This aligns with previous research which also suggests that upbringing can influence current eating habits (Lake and Townshead, 2006). It would seem prudent to include family and whanau into any nutritional education which is provided to development players, especially as previous research into childhood nutritional intakes has shown parents and family members are influencers to healthy eating (Birch et al., 2007; Emmett and Jones, 2015). Foods which are affordable must be included in any education program for players at all levels, especially where players are not full time professionals. A point which is highlighted by the considerable agreement amongst participants from families greater than five (over 50% of the study population) that they perceived they had unhealthy eating habits and dietary intakes during childhood which were restricted by food costs. Similar statements about financial factors governing current food choices were also seen amongst non-super rugby players. However, education can only go so far and if there is insufficient money to buy food then nutrition knowledge will have limited benefit.

Access to healthy food and the increasing cost of healthy food is well-recognised among professionals as a barrier to optimal nutritional intake (Heaney, 2011). Elite athletes on a limited budget report that financial constraints interfere with making food choices that support a healthy diet (Heaney, 2011). In the current study, food costs in childhood and adulthood were barriers to optimal nutritional intake. Food cost has been reported as influencing dietary intakes in male collegiate football players (Long et al., 2011). Club provisions of snacks and meals or supermarket sponsorship could be strategies to promote desired dietary intakes. However, providing food for the player without provision for the whanau (family), is unlikely to solve the issue as players will likely share the food meaning provision is still sub-optimal. The issue probably needs a wider scope including public health policy around food insecurity of the population. For example, the provision of meals in schools could potentially aid with food provision of developing players but the wider community, as well as serve as an opportunity for nutrition education (Oostindjer et al., 2017).

The impact of media on nutritional intakes in the current study demonstrated differences by playing level with media not influencing intakes amongst the Super Rugby players, who instead relied on the team nutritionist, whereas those at lower levels, provincial and developmental reported being influenced by media and team mates something which has previously been reported amongst adolescent rugby players (Stokes et al., 2018). Although nutritional support at levels below Super Rugby is likely limited by funding this finding does highlight a potential need for education regarding the evaluation of information seen online or heard via team mates to ensure nutritional requirements and consequently health and performance are not compromised. If rugby players, before they gain a professional contract, do not have access to professional nutritional advice; they are potentially vulnerable to opinions and unsubstantiated claims via social media, which could impact their development as players and potentially harm their future careers. The potential of social media to compromise health and performance is something which was also highlighted amongst adolescent rugby players by Stokes et al. (2018).

Body composition was raised as both a barrier and enabler to optimal dietary intake. Those with unfavourable body composition tended to have dietary behaviours that led to the overconsumption of food and/or food choices which weren't aligned with the nutritional guidelines. These dietary behaviours appear to be related to a lack of organisation and planning of meals throughout the week, this does suggest that assisting players to become more organised about meal preparation or delivery of meals could be of benefit to ensuring body composition goals are attained. Lack of time and convenience have been reported as factors in food choice for athletes (Smart and Bisogni, 2001; Heaney, 2011; Long et al., 2011; Birkenhead and Slater, 2015; Stokes et al., 2018; Bentley et al., 2021). Lack of meal planning and daily food shopping could increase the likelihood of consuming high fat high sugar processed foods, as in the general population meal planning has been associated with a healthier diet and lower levels of obesity (Ducrot et al., 2017). Time spent training and the weekly competition schedule mean that athletes have a lack of time to purchase, prepare and manage their dietary choices (Long et al., 2011). Athletes value foods that are convenient and easy to prepare. Often this leads to athletes purchasing convenience foods, which are typically unhealthy and not optimal for athletes (Birkenhead and Slater, 2015). This was more commonly seen amongst developmental players who were also working or studying alongside training in order to support themselves and families. This indicates the need for nutritional time management education, including quick cooks, healthy takeaway choices and meal prepping within sporting environments. Experienced professional players from similar backgrounds to the developmental players e.g., playing position, ethnicity, background, could act as mentors. This may help younger players with their dietary choices by providing advice on negotiating the barriers to optimal dietary intakes, such as organisational skills and importance of nutrition for performance and the use of social media. Although the selection and training of the mentor would be important to ensure the advice provided will be of benefit to the mentee.

Body composition was also an enabler for optimising dietary intakes through its influence on performance. Participants identified body composition as an enabler to optimise nutrition strategies in order to reach body composition goals and therefore perform at a higher level. Similar findings have been reported in previous research whereby sports performance has been reported by athletes as a motivation to food choice (Heaney, 2011; Stokes et al., 2018). Therefore, focusing on the performance benefits of food choices may enable players to make adequate dietary choices and could be built into education programs alongside weekly meal planning.

Despite the findings of the current research they must be interpreted with caution as the Super Rugby players all played for the same club, with access to a nutritionist. However, some of these players had previously played for other franchises. Furthermore, although the current study included players from a range of ethnicities and socio-demographic backgrounds further research should be conducted to further investigate these potential influencers. Investigations into the influences on dietary intakes of female rugby players should also be undertaken to aid the delivery of nutritional education to this group of players. Conducting individual interviews could be seen as a strength of the study as it removes any potential peer-pressure with responses, however, the study does rely on the assumption participants responded honestly.

## Conclusion

This study uniquely describes the influences on dietary intakes amongst elite Rugby Union player's. It highlights that nutritional interventions require consideration of many social and economic factors which influence players' ability to adhere to any dietary intervention. For example, organisational skills appear to be associated with maintenance of body composition and could be a non-nutrition skill that may help athletes adhere to dietary advice. Finally, social media influences dietary intakes more amongst developmental rugby players than super rugby players, thus educating players early about interpreting such information is recommended.

## Data Availability Statement

The datasets presented in this article are not readily available due to the nature of this research, participants of this study did not agree for their data to be shared publicly, so supporting data is not available. Requests to access the datasets should be directed to the corresponding author.

## Ethics Statement

The studies involving human participants were reviewed and approved by the University of Otago Human Ethics and the University of Stirling Ethics Committees. Written informed consent from the participants' legal guardian/next of kin was not required to participate in this study in accordance with the national legislation and the institutional requirements.

## Author Contributions

DB and KB were responsible for the study design, data collection, data analysis, and critically reviewing the manuscript. AS was involved with data collection and undertook data collection and analysed the data as well as drafting the manuscript. SG and BS were involved with study design, data analysis, and critically reviewing the manuscript. All authors approved the final version of the paper.

## Funding

This study was funded by the Department of Human Nutrition, University of Otago.

## Conflict of Interest

The authors declare that the research was conducted in the absence of any commercial or financial relationships that could be construed as a potential conflict of interest.

## Publisher's Note

All claims expressed in this article are solely those of the authors and do not necessarily represent those of their affiliated organizations, or those of the publisher, the editors and the reviewers. Any product that may be evaluated in this article, or claim that may be made by its manufacturer, is not guaranteed or endorsed by the publisher.
